# Editorial: Emerging technologies for viability enumeration of live microorganisms

**DOI:** 10.3389/fmicb.2024.1546438

**Published:** 2025-01-16

**Authors:** Hanan R. Shehata, Marco Pane, Elna M. Buys, Binu Koshy, Christina S. Vegge, Jean L. Schoeni

**Affiliations:** ^1^Purity-IQ Inc., Guelph, ON, Canada; ^2^Probiotical Research S.r.l., Novara, Italy; ^3^Department of Consumer and Food Science, University of Pretoria, Hatfield, South Africa; ^4^Dietary Supplements and Herbal Medicines, United States Pharmacopeia, Rockville, MD, United States; ^5^Research & Development, Probi AB, Lund, Sweden; ^6^Eurofins Microbiology, Inc., Madison, WI, United States

**Keywords:** emerging technologies, viability enumeration, potency, live microorganisms, beneficial organisms, probiotics, live biotherapeutic products, microbiome products

## Introduction

The live microorganism industry is rapidly growing, producing probiotics and live biotherapeutic products (LBPs) designed to deliver health benefits. Ensuring these products contain viable, strain-specific microorganisms at effective levels is essential, but accurately measuring viability and potency remains challenging.

Colony-forming unit (CFU) enumeration, the traditional gold standard, relies on a cell's ability to form colonies on culture media. While widely used, it has significant limitations. CFU methods fail to account for viable but non-culturable (VBNC) cells, which maintain metabolic activity but cannot grow on culture media. Moreover, CFU enumeration often falls short for probiotic blends, as strains with varying growth requirements or interactions may not form colonies under standardized conditions.

With growing consumer awareness and stricter regulatory demands, more accurate and comprehensive enumeration techniques are needed. Emerging methods such as flow cytometry, real-time PCR, digital PCR, and advanced imaging assess viability based on cellular activity rather than replication alone. These approaches offer reliable assessments of complex probiotic formulations, ensuring higher product quality and efficacy.

Adopting advanced techniques is critical to meet regulatory standards, enhance product reliability, and build consumer trust, marking a significant step forward in ensuring the health benefits of live microorganism products.

“*Emerging Technologies for Viability Enumeration of Live Microorganisms*,” focuses on advanced techniques and how researchers have adapted them to fit their needs. The Research Topic includes two reviews, five reports detailing successful development and use of real-time PCR (qPCR) assays for probiotics, two articles highlighting the adaptability of flow cytometry, one extending understanding of microbial activity and viability using isothermal microcalorimetry, and one utilizing Cell Counting Kit-8.

## Reviews

Boyte et al. reviewed available enumeration methods for probiotics and postbiotics, including plate counting (culture dependent) and alternative, culture-independent methods: flow cytometry, real-time PCR (qPCR), and digital PCR (dPCR). Advantages, limitations, viability determination, and the potential of each technique for use in the probiotics industry, including newer categories such as next generation probiotics and tyndallized/heat-killed bacteria were discussed.

Noting that maintaining cell viability is essential to the therapeutic functionalities of probiotic foods, Sibanda et al. reviewed viability challenges encountered from manufacturing through consumption of fermented dairy foods. The authors emphasized the critical nature of viability enumeration for quality assurance and discussed flow cytometry, propidium monoazide-quantitative polymerase chain reaction (PMA-qPCR), next-generation sequencing, and single-cell Raman spectroscopy (SCRS) approaches to reduce quality assurance challenges.

## Polymerase chain reaction

Several PCR based methods were developed for the enumeration of probiotic targets. Four articles described the development of eight species-specific enumeration methods and one article described the development of one strain-specific enumeration method. Researchers successfully demonstrated that PMA-qPCR could be used for species-specific viability enumeration of well-known probiotics: *Lactobacillus acidophilus* and *Bifidobacterium bifidum* (Catone et al.), *Lactobacillus delbrueckii* subsp. *bulgaricus, Streptococcus thermophilus*, and *Bifidobacterium* spp. (Marole et al.), *Lacticaseibacillus rhamnosus* (Marole et al.; Guo et al.), and *Lacticaseibacillus paracasei* (Guo et al.). All methods showed PMA efficiently inhibited counting dead cells and were highly specific to target species. These methods were applied to various matrices. This demonstrates the flexibility of PCR and makes the collection useful to those working in research and development, quality assurance, and manufacturing.

Shehata et al. developed a strain-specific PMAxx-qPCR method for strain *B. longum* subsp. *longum* UABl-14. High specificity, reaction efficiency, and precision were demonstrated. The method enabled stability monitoring of the target strain in multi-strain finished products during storage, which cannot be achieved using plate count methods.

## Flow cytometry

Jordal et al. conducted a ring test for fluorescence flow cytometry (FCC) and a study comparing the ring test results to those of impedance flow cytometry (IFC) to address challenges presented by traditional plate counting methods. It appears to be the first peer-reviewed comparison of FCC and IFC. Both methods evaluate the presence of intact membranes for single cells in solutions. The FCC ring test demonstrated robustness across changes in equipment, procedures, materials, and operators. After a one-time per strain optimization, the IFC method showed good agreement with FCC results. Combined, the ring test and comparison results indicated that these culture-independent flow cytometry methods saved time, were reliable, precise, adaptable to bacterial enumeration, and allowed exploration of viability.

A 2.0–2.5 h flow cytometry and fluorescence *in situ* hybridization (Flow-FISH) protocol specific for Gram-positive bacteria in probiotic products was presented by Snaidr et al.. Individual probiotics and three-species blends were evaluated by Flow-FISH protocol alone or in combination with live/dead (L/D) staining and/or plate counting. Data showed: (1) Flow-FISH and L/D staining outperformed standard plate counting in quantification. (2) Flow-FISH surpassed plate counting and L/D staining in repeatability and uncertainty. (3) Unlike plating and staining, Flow-FISH was capable of species-specific quantification in blended products. (4) Flow-FISH performed linearly and demonstrated robustness between two flow cytometry instruments. The authors suggested their study established the use of Flow-FISH for comprehensive quality control.

## Isothermal microcalorimetry

ICM captures changes in heat produced by living organisms (e.g., metabolic processes). Morazzoni et al. contributed a proof-of-concept study featuring the application of IMC to determine viability and growth dynamics and its correlation to the plate counts for *Lacticaseibacillus rhamnosus* and *Limosilactobacillus fermentum*. Experiments established suitability of ICM for viability assessment and enumeration of probiotic products. Relationships between ICM and plate counting were determined via standard curves and linear regression analyses. Method robustness was observed through the maintenance of correlations between time-to-peak (TTP) heat detected in ICM and CFU/mL from plate counting across various culture conditions. Finally, IMC, flow cytometry, and acidification measurement experiments were conducted under diverse conditions to demonstrate how IMC can be used as a complementary approach that extends understanding of microbial activity and viability.

## Cell counting Kit-8

Yang et al. (2021) introduced the application of tetrazolium-based colorimetric cell counting kits (CCK-8) to live bacteria. Health and clinical scientists have adopted CCK-8 to enumerate viable probiotics (Chang et al., 2024; Sudan et al., 2022; Xu et al., 2023; Yue et al., 2022). Here, Shang et al. investigated the role of *B. longum* in the prevention and treatment of colorectal cancer (CRC). CCK-8 was used to optimize the concentration of viable *B. longum* cells and time of coculturing with CRC. The optimized conditions were applied to various assays to demonstrate the inhibitory effects of *B. longum*.

## Conclusions

This Research Topic highlights the need to improve viability enumeration methods for live microorganisms. The enumeration technologies presented have provided innovative approaches for the enumeration of live microorganisms that are faster, with higher specificity and precision, and lower uncertainties than plate counting. These are characteristics needed in research, manufacturing, and clinical settings. As innovations are adopted, new insights and understanding generated will drive improvements from product conception to consumer confidence.
